# BRIDGES: a patient-centered, community-informed HIV doula program that supports infant feeding choice among Hispanic and Caribbean families: a study protocol

**DOI:** 10.3389/frph.2026.1896729

**Published:** 2026-08-28

**Authors:** Rachel Mpanu-Mpanu, Melanie Francis, Kassandra N. Garcia, Lunthita M. Duthely

**Affiliations:** 1 Tamer Family Department of Obstetrics, Gynecology and Reproductive Sciences, University of Miami School of Medicine, Miami, FL, United States; 2 Ross University School of Medicine, Bridgetown, Barbados; 3 Department of Public Health Sciences, University of Miami School of Medicine, Miami, FL, United States

**Keywords:** breastfeeding, doula, HIV, infant feeding, maternal and child health

## Abstract

**Background:**

In 2023, U.S. Perinatal HIV Guidelines shifted from recommending exclusive formula feeding to endorsing shared decision-making for infant feeding among pregnant and postpartum persons with HIV (PPWH) on antiretroviral therapy (ART) with sustained viral suppression. While the risk of HIV transmission through breastfeeding is small (< 1%) with sustained viral suppression, adherence protocols are complex and clinical support infrastructure remains underdeveloped. Doula care has demonstrated benefits for breastfeeding initiation, maternal anxiety reduction, and postpartum follow-up, yet no studies have examined HIV-specialized doula programs supporting infant feeding choice among non-English speaking PPWH in North America.

**Objective:**

Using a patient-centered, community-informed framework, BRIDGES (BReast and Infant feeding Doulas to address Gaps in Education and Support) is an HIV Doula education and support program designed to improve maternal/child health outcomes and reduce perinatal transmission. The program aims to support heterogeneous infant feeding options through doula-mediated prenatal education, ART adherence support, lactation counseling, and postnatal care coordination.

**Methods:**

In collaboration with the Pediatric Community Advisory Board, South Florida Health Departments' Perinatal HIV Programs, and University of Miami (UM) Ob/Gyn, a doula-based intervention was developed for pregnant and postpartum women with HIV. The study employs a sequential mixed-methods design: Aim 1 builds the BRIDGES intervention through community engagement, embedding an Infant Feeding Choice Community Advisory Group; Aim 2 evaluates BRIDGES in a single-arm interventional study of 15 mother–baby dyads. Data collection includes sociodemographic, clinical, obstetrical, and psychosocial measures (HIV stigma, medical mistrust, resilience), along with individual and group interviews. The Consolidated Framework for Implementation Research (CFIR) guides evaluation of implementation determinants.

**Results:**

The BRIDGES intervention pairs each mother–infant dyad with an HIV-trained doula from enrollment during pregnancy through 12 weeks postpartum. The doula supplements clinic education with prenatal education on infant feeding options (breastfeeding, formula, banked donor milk), ART adherence support, lactation counseling, and guidance on clinical monitoring protocols specific to feeding choice. Clinical monitoring protocols differentiate between breastfeeding dyads (monthly maternal viral load, infant ARV prophylaxis until weaning, enhanced infant HIV testing) and formula-feeding dyads (standard of care).

**Significance:**

BRIDGES addresses a critical gap at the intersection of evolving HIV infant feeding guidelines and doula-supported perinatal care. This intervention was shaped by a collaborative effort that ensures its cultural relevance, linguistic inclusivity, and alignment with community needs, amplifying its potential impact on supporting infant feeding choice among PPWH. Findings may inform other U.S.-based HIV clinics providing pregnancy care services to PPWH.

**Clinical Trial Registration:**

Identifier: NCT07664423. https://clinicaltrials.gov/study/NCT07664423.

## 1 Introduction

Each year, approximately 3,500–5,000 women with HIV give birth in the United States (1–3). Through universal prenatal HIV screening, combination antiretroviral therapy (ART), and until recently complete avoidance of breastfeeding, the perinatal HIV transmission rate declined from approximately 25% in the pre-ART era to 0.9% by 2019, achieving the national elimination goal of less than 1% (1, 3, 4). A 2025 systematic review and meta-analysis of approximately 80,000 mother–child pairs found zero observed perinatal transmissions among women who started ART before conception and maintained a viral load below 50 copies/mL through delivery (5). This success, however, came at a cost: the blanket recommendation against breastfeeding failed to account for the immunologic, nutritional, bonding, and cardiometabolic benefits of human milk for both mother and infant (6, 7), and disproportionately affected Black, Hispanic/Latino, and immigrant communities for whom breastfeeding carries deep cultural significance and for whom breastfeeding disparities are already pronounced. Breastfeeding initiation among Black infants (74.5%) lags substantially behind White (85.3%) and Hispanic (86.8%) infants nationally (8, 9).

In 2023, the U.S. DHHS Perinatal HIV Guidelines were updated to endorse shared decision-making for infant feeding among PPWH on ART with sustained viral suppression, acknowledging that the risk of postnatal HIV transmission through breastfeeding is very low, up to 1%, though not zero (6, 10, 11). The American Academy of Pediatrics (AAP) 2024 Clinical Report and the Infectious Diseases Society of America (IDSA) 2024 primary care guidance followed with concordant recommendations (7, 12). Yet standardized clinical infrastructure to deliver this new paradigm consistently across sites remains limited, even as some individual programs have developed local procedures to support shared decision-making. No data inform the optimal frequency of viral load monitoring during breastfeeding; no data exist on postnatal transmission risk with mixed feeding while on ART; infant ARV prophylaxis strategies vary from no prophylaxis to three-drug regimens through weaning with no consensus; and only an estimated 184–214 breastfeeding infants with perinatal HIV exposure have been described across observational studies in high-income settings, none designed to evaluate transmission (5, 10, 11). A 2025 meta-regression updating global vertical transmission probability estimates for the UNAIDS Spectrum-AIM model found that breastfeeding transmission probabilities were revised upward compared to prior default values, further underscoring the need for structured support and monitoring for breastfeeding PPWH (13).

Adherence protocols are complex: PPWH who breastfeed must maintain 100% ART adherence, undergo enhanced maternal and infant viral load monitoring, manage breastfeeding complications (mastitis, cracked nipples, infant thrush) that may warrant temporary interruption, and navigate weaning all while contending with HIV stigma, medical mistrust, and postpartum depression, which occurs more frequently among individuals with HIV (7, 10, 11). A survey of U.S. HIV specialty providers found that one-third were aware that patients breastfed after being advised not to do so, underscoring the urgent need for structured support (11).

Successful breastfeeding among PPWH depends not only on patient autonomy but also on access to accurate, evidence-based education and comprehensive clinical support. Challenges may be particularly pronounced among marginalized populations, which often face disproportionate barriers within the U.S. healthcare system. In a qualitative study of a predominantly multicultural population in South Florida, breastfeeding was viewed as an important cultural and familial practice, yet barriers caused mainly by misinformation prevented informed infant-feeding decisions (14). Among participants, 45% identified fear of HIV transmission through breastfeeding as a barrier, while 25% reported experiencing societal and cultural pressures related to infant-feeding practices (14). These concerns may influence infant-feeding decisions, impacting both evidence-based feeding practices and neonatal health. Given the multitude of intersectional factors influencing infant-feeding practices, additional support should be directed toward developing culturally responsive interventions that address the barriers faced by women living with HIV.

Doula care has demonstrated consistent benefits for breastfeeding initiation, maternal anxiety reduction, and postpartum follow-up. A 2026 systematic review of 21 clinical trials found doula care most consistently associated with improved breastfeeding initiation and reduced maternal anxiety (15), and a large retrospective cohort study (*N* = 17,831) demonstrated 20% higher exclusive breastfeeding rates with doula support, with effects consistent across race and insurance status (16). Doulas have been identified as a promising strategy to mitigate racial disparities in maternal-infant outcomes (17–19). Beyond improving clinical outcomes, doulas provide culturally appropriate care and informational support that may help address structural barriers. Research has shown that doula support is associated with a 25% reduction in cesarean deliveries and reduced rates of preterm birth among historically marginalized populations (20). A retrospective cohort study of deliveries recorded from January 2021 to December 2022 found that patients with doula care experienced more frequent vaginal birth after C-Section (VBACs), fewer preterm and early preterm deliveries, fewer indicated preterm deliveries, and higher rates of exclusive breastfeeding (18). In the postpartum period, individuals who delivered under doula care were also more likely to continue and engage with postnatal care within 6 weeks of delivery. Although that study did not specifically include PPWH, the findings align with the key components required for successful breastfeeding in this population, including sustained engagement in care, adherence to ART for both mother–infant dyads, maintenance of viral suppression, and consistent postpartum monitoring.

As infant-feeding recommendations increasingly emphasize shared decision-making, doulas may serve as valuable members of the perinatal care team by providing culturally competent care and supporting informed infant-feeding decisions. However, no published studies have evaluated HIV-specialized doula programs in the United States. The Kotawêw study in Canada is the sole published work examining doula care within the HIV care continuum, describing Indigenous doulas as providing peer mentorship, practical HIV education, stigma reduction, and structural advocacy (21). BRIDGES (BReast and Infant feeding Doulas to address Gaps in Education and Support) is the first U.S. HIV-specialized doula intervention supporting infant feeding choice. Conducted at the University of Miami (UM) Department of Obstetrics, Gynecology and Reproductive Sciences which has cared for more than 4,000 PPWH in South Florida, a region with among the highest rates of perinatal HIV in the nation (4, 22) this exploratory pilot study employs a sequential mixed-methods design and aims to: (1) build a patient-centered, community-informed HIV doula program that supports infant feeding choice while maintaining viral suppression, informed by an Infant Feeding Choice Community Advisory Group, with qualitative analysis of Advisory Group input guiding intervention development; (2) evaluate the feasibility and acceptability of BRIDGES in a single-arm interventional study of 15 mother–baby dyads with heterogeneous feeding intentions, including qualitative analysis of participant interviews; and (3) generate preliminary data to inform a future multi-site trial generalizable to U.S.-based HIV clinics providing pregnancy care to PPWH.

## 2 Methods and analysis

### 2.1 Development of study team to address community needs

A multidisciplinary team will inform the BRIDGES study, bringing together HIV clinicians, obstetricians, pediatricians, doulas, implementation scientists, and community members with lived experience of HIV, including women with HIV who have been pregnant or postpartum, to ensure that input on intervention design reflects the perspectives of the women the program is intended to serve. The collaborative was built upon the belief that to address community needs, interventions must be designed and developed not only for the community, but with the community. The study team includes the UM Women's HIV Clinic, the UM Center for AIDS Research (CFAR) Clinical Research Unit, and South Florida Health Departments' Perinatal HIV Programs.

Central to this approach is the establishment of an Infant Feeding Choice Community Advisory Group, embedded within the existing Pediatric Community Advisory Board. This Advisory Group (maximum 20 members) will be recruited from the Pediatric HIV Community Advisory Board and will include men and women ≥18 years old who have experience working with or are living with HIV, including women with HIV who have lived experience of infant feeding decision-making, and are able to communicate at the 6th grade level in English, Spanish, or Haitian Creole. The Advisory Group will also include several members with personal birthing and infant feeding experience, even if not living with HIV, as dual lived experience of HIV and infant feeding is considered especially valuable. The Advisory Group will meet twice initially to establish the board, then once every 6 weeks thereafter, with meetings lasting approximately one hour. Advisory board notes will be taken, de-identified, and analyzed qualitatively for research purposes. Advisory Group members will not receive financial compensation, consistent with their role as advisors rather than research participants. This structure ensures that the intervention is informed by the lived experiences of people with HIV and the expertise of perinatal HIV providers, consistent with best practices for community advisory boards in HIV research (10, 12).

### 2.2 Theoretical frameworks

BRIDGES is grounded in two complementary frameworks. First, the Consolidated Framework for Implementation Research (CFIR) guides evaluation of implementation determinants across five domains: intervention characteristics, outer setting, inner setting, characteristics of individuals, and process (23, 24). CFIR has been successfully applied to evaluate prevention of vertical (perinatal) HIV transmission (PMTCT) programs (25), where it identified key constructs distinguishing high- and low-performing facilities, including networks and communication, available resources, external change agents, executing, and reflecting and evaluating. In the BRIDGES study, CFIR will serve as both a planning tool during intervention development (Aim 1) and a deductive coding framework during qualitative analysis (Aim 2).

Second, a patient-centered, shared decision-making model consistent with the 2023/2025 U.S. Perinatal Guidelines and the 2024 AAP Clinical Report anchors the intervention's clinical approach. This shared decision-making approach is consistent with current U.S. perinatal HIV infant-feeding guidelines (7, 11). This model emphasizes nonjudgmental counseling, exploration of motivations and barriers, and support for autonomous infant feeding decisions (7, 10, 11). The doula serves as the primary vehicle for operationalizing shared decision-making, providing sustained, culturally concordant support that bridges clinical recommendations and individual values, preferences, and feeding plans.

### 2.3 Study design and setting

The development of this clinical trial, from the study design to the protocols, is an iterative process that will involve extensive feedback from the study team, Advisory Group, and clinical partners.

**Study design:** This is an exploratory, single-arm, mixed-methods interventional study employing a sequential design in which Aim 1 (community engagement and intervention development) informs the implementation of Aim 2 (interventional study). The protocol will be registered on ClinicalTrials.gov prior to enrollment.

**Study setting:** All study activities will take place at the UM Women's HIV Clinic and the UM CFAR Clinical Research Unit in Miami-Dade County, Florida. Study procedures may also be conducted via Zoom for Healthcare. South Florida represents an ideal setting: the region has among the highest rates of HIV transmission in the United States. An analysis of 4,337 singleton births with perinatal HIV exposure reported to the Florida Department of Health found a 1.6% perinatal transmission rate, with late maternal HIV diagnosis as the primary driver of missed prevention opportunities (22). The population is racially, ethnically, and linguistically diverse, with significant representation of communities disproportionately affected by HIV, including Black, Hispanic/Latina, and Haitian Creole-speaking populations.

### 2.4 Participants

**Advisory Group members (Aim 1):** Men and women ≥18 years old who have experience working with or are living with HIV and are able to communicate at the 6th grade level in English, Spanish, or Haitian Creole. Maximum of 20 members.

**Patient participants (Aim 2):** Inclusion criteria are women ≥18 years old with a documented HIV diagnosis; currently pregnant or postpartum; residing in South Florida; and able to communicate at the 6th grade level in English, Spanish, or Haitian Creole. Exclusion criteria are persons not able to verbally consent in English, Spanish, or Haitian Creole. There is no maximum age. Target enrollment is *N* = 15 mother–baby dyads.

Women who are virologically suppressed throughout pregnancy may also enroll after delivery and receive the postnatal component of the intervention; enrollment timing (prenatal versus postnatal) will be recorded to account for differential exposure to intervention components.

### 2.5 Intervention: the BRIDGES HIV doula program

The BRIDGES intervention pairs each enrolled mother–infant dyad with an HIV-trained doula (study team personnel) who provides the following services from enrollment during pregnancy through 12 weeks postpartum:

- Prenatal education on infant feeding options (breastfeeding, formula, banked donor milk), consistent with the shared decision-making framework recommended by U.S. Perinatal Guidelines (10, 11)
- ART adherence support, including medication reminders, barrier identification, and problem-solving
- Appointment adherence support to ensure attendance at prenatal, postnatal, and pediatric visits
- Lactation counseling and support for mothers who elect to breastfeed, including education on exclusive breastfeeding recommendations, breast health monitoring, and weaning guidance
- Guidance on adherence protocols specific to infant feeding choice, including infant ARV prophylaxis schedules and maternal/infant viral load monitoring

The doula will meet with mothers and their babies during regularly scheduled prenatal, postnatal, and pediatric visits, either before or after the scheduled clinical visit either in person or remotely. Each visit will take up to 60 min. Postnatally, mothers and infants will meet with the doula at each postnatal visit (one or two) and every 4 weeks for 12 weeks.

The clinical monitoring schedule for HIV-exposed mother–infant dyads below follows guideline-based care; the doula-mediated education, adherence support, and care coordination delivered through BRIDGES are not standard of care and represent the adapted, novel component of the intervention.

Clinical monitoring protocols are differentiated by feeding choice. For mothers who initiate breastfeeding: mothers will continue ART throughout breastfeeding, with the goal of maintaining HIV viral load < 50 copies/mL; infants will receive ARV prophylaxis until weaned off breast milk; maternal HIV viral load will be monitored monthly for up to 3 months or until the infant is weaned, consistent with the guideline recommendation of monitoring every 1–2 months; infant HIV nucleic acid testing will follow the schedule recommended by the AAP (at 14–21 days, 1–2 months, 4–6 months, every 2 months during lactation, and at 4–6 weeks and 3 and 6 months after weaning); breastfeeding will be temporarily interrupted or discontinued if maternal viral load becomes detectable, particularly if ≥200 copies/mL; and the doula will educate on and monitor for breastfeeding complications (mastitis, cracked/bleeding nipples, infant thrush) that may warrant temporary breastfeeding interruption (6, 7, 10, 11). For mothers who elect to formula feed: standard of care will be followed, with infant ARV prophylaxis for 4 weeks and viral load measured at birth, 2 weeks, and 4 weeks.

For mothers who breastfeed, expressed-milk pumping and pasteurization (including exclusive pumping) will be offered as a supported option during viral blips, mastitis, or other periods of increased transmission risk for those who prefer not to introduce formula. Infant antiretroviral prophylaxis will be provided for the duration of breastfeeding per site protocol, and banked donor milk (including use as a supplement to the mother's own milk) will be supported, with data collected on source and frequency of use. Specific infant ARV regimen and prophylaxis decisions will follow the treating clinical team's guidance and institutional protocol.

The clinical monitoring protocols follow guideline-based standard of care and are fixed; the doula-delivered intervention components (educational content, materials, and delivery approach) and recruitment procedures are co-developed and iteratively refined with the Advisory Group and clinical partners. The Advisory Group structure will be established within the existing Pediatric Community Advisory Board framework. Participants will be enrolled during pregnancy regardless of infant feeding intention, allowing the study to capture the full spectrum of infant feeding decisions and the doula's role in supporting each choice.

### 2.6 Study procedures overview

**Figure 1:**
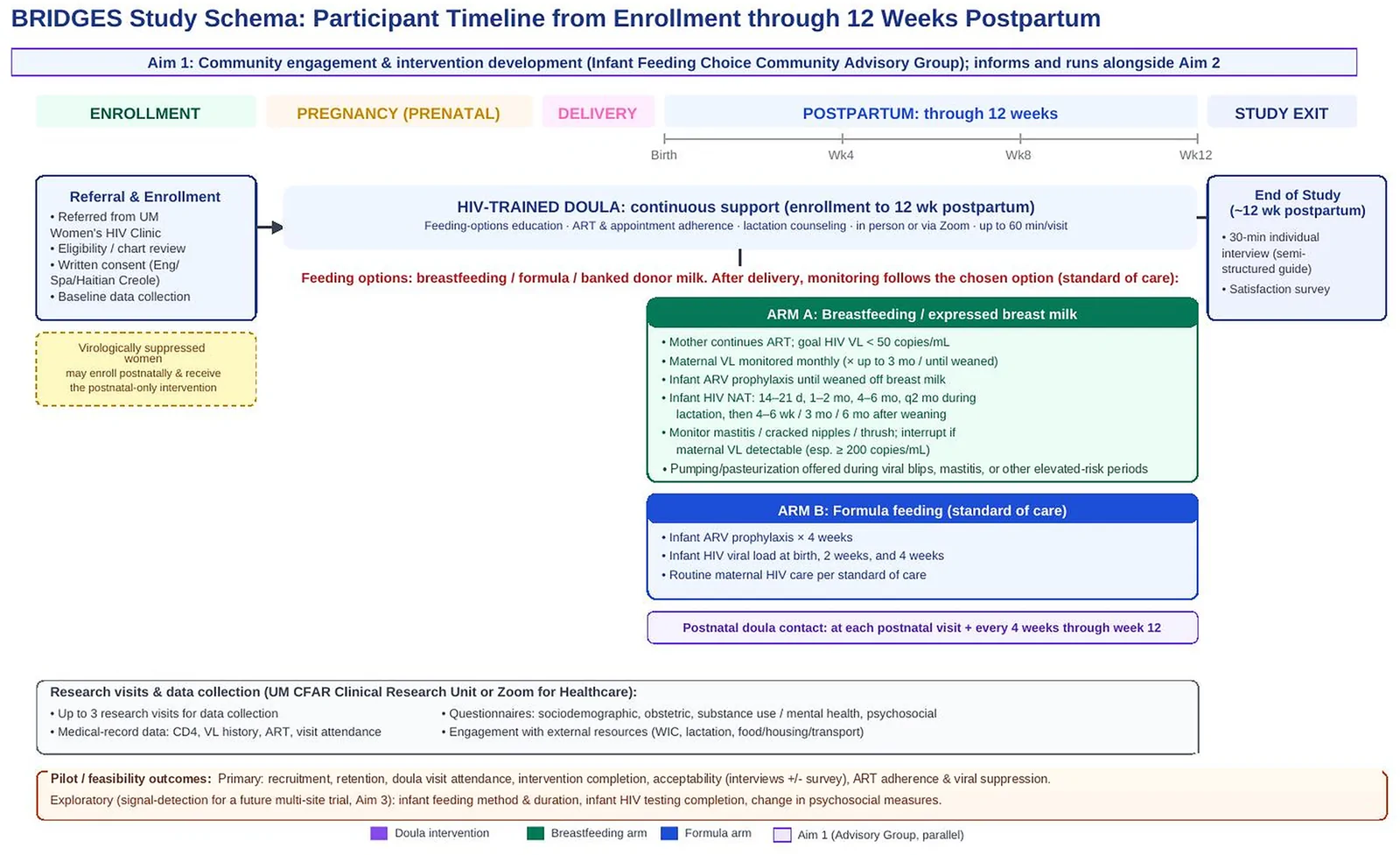
BRIDGES study schema: participant timeline from enrollment through 12 weeks postpartum.

Figure 1 summarizes the participant sequence from enrollment through 12 weeks postpartum. Eligible women are referred from the UM Women's HIV Clinic and enrolled during pregnancy at the UM CFAR Clinical Research Unit, where written informed consent and baseline data collection occur. From enrollment through 12 weeks postpartum, each mother–infant dyad is paired with an HIV-trained doula who provides feeding-options education, ART and appointment adherence support, and lactation counseling, in person or via Zoom for Healthcare. After delivery, clinical monitoring follows the mother's infant-feeding choice (breastfeeding/expressed milk versus formula), using the guideline-based schedules described in Section 2.5. Participants complete up to three research visits for questionnaire and medical-record data collection, with postnatal doula contact at each postnatal visit and every four weeks through week 12, and a 30-minute individual interview at study exit (approximately 12 weeks postpartum).

### 2.7 Data collection

All quantitative questionnaires will be available in English, Spanish, and Haitian Creole. These include:

- Sociodemographic Questionnaire: Gathers data on participants' sociodemographic information (age, race/ethnicity, education, income, insurance status, language), overall health, and pregnancy-related factors.
- HIV Health-Related Data: Self-reported items on overall health and HIV history (collected by questionnaire).
- Medical Record Data: Extracted from the electronic medical record (not by questionnaire): CD4 count, viral load history, ART regimen and duration, and attendance at prenatal, postnatal, and pediatric visits.
- Obstetrical History: Gravidity, parity, prior infant feeding experiences.
- Substance Use and Mental Health History: Including screening for postpartum depression using the Edinburgh Postnatal Depression Scale (EPDS), which occurs more frequently in individuals with HIV compared to those without HIV (10, 11).
- Psychosocial Measures: HIV stigma measured with the 40-item HIV Stigma Scale (HSS) (26), medical mistrust measured with the 12-item Group-Based Medical Mistrust Scale (GBMMS) (27), and resilience measured with the 25-item Connor-Davidson Resilience Scale (CD-RISC-25) (28).
- Engagement with External Resources: Use of and referral to external support services, including the Special Supplemental Nutrition Program for Women, Infants, and Children (WIC), lactation consultation, food assistance, housing, and transportation services, will be documented at each doula visit to characterize the broader support context surrounding each dyad.
- Infant Feeding Outcomes: Feeding method chosen, duration of breastfeeding if applicable, breastfeeding complications, and, for participants using banked donor milk, the source, frequency, and whether donor milk is used exclusively or as a supplement to the mother's own milk.
- ART Adherence Measures: ART adherence will be assessed using the 3-item self-report adherence measure (Wilson et al.) (29), a 30-day count of days with a missed dose plus self-ratings of how well and how often medications were taken as prescribed, together with maternal HIV viral load from the electronic medical record as a biological marker of sustained adherence.
- Maternal and Infant HIV Viral Load Results and Infant HIV Testing Results.
- Doula Visit Attendance and Engagement: Visit engagement will be operationalized as the proportion of scheduled doula visits attended (number of completed visits divided by the number scheduled per the protocol), the proportion of recommended prenatal, postnatal, and pediatric clinical visits attended, and doula-documented participation during each contact. These attendance and engagement metrics constitute the primary feasibility outcomes.
- Participant Satisfaction: Participant satisfaction will be assessed using a brief validated instrument, the Client Satisfaction Questionnaire (CSQ-8), administered at study completion to complement the qualitative interviews.

In addition to doula visits, participants will meet with research staff for up to 3 additional study visits at the UM CFAR Clinical Research Unit for data collection.

Qualitative data collection includes individual interviews. After completing visits with the doula (up to 12 weeks postpartum), patient participants will be invited to participate in a 30-minute individual interview to share their experiences with the BRIDGES study. Interviews will be conducted at the UM CFAR Research Unit or via Zoom for Healthcare. A semi-structured interview guide will explore experiences with the doula intervention, perceived impact on infant feeding decision-making and confidence, barriers and facilitators to ART adherence during the perinatal period, experiences with breastfeeding complications (if applicable), and suggestions for intervention improvement. To ensure data sources are not conflated, participant interviews (Aim 2) will use an individual format; group formats are reserved for the Advisory Group (Aim 1).

During Advisory Group meetings (Aim 1), individual and group interview data will be gathered using semi-structured guides. Data will inform the adaptation and refinement of the BRIDGES intervention.

### 2.8 Recruitment and enrollment

UM HIV providers (non-study team members) will be made aware of the study during weekly meetings and grand rounds and will be given information to share with their patients to contact the clinical research team located in the UM CFAR Clinical Research Unit. Participants will be referred from the UM Women's HIV Clinic to the UM CFAR Clinical Research Unit, where enrollment activities will take place. Medical records may be reviewed for inclusion/exclusion criteria. The study will be open to all women who are referred, regardless of infant feeding intention (e.g., breastfeeding, formula feeding). The primary sources of referral will be University of Miami HIV care providers listed on the protocol as study team members.

Upon confirming eligibility, participants will undergo the consent process and be enrolled. Written consent will be obtained in a private room at the UM CFAR Clinical Research Unit. The research assistant will inform the women of the study's purpose and protocol and read the informed consent, which outlines all aspects of the study. If a participant wants to discuss participation with family members, the research assistant will schedule another appointment. Consent will be obtained in English, Spanish, and Haitian Creole.

Advisory Board members will be recruited from the Pediatric HIV Community Advisory Board and will provide verbal consent at the first Advisory meeting.

### 2.9 Outcomes

As an exploratory pilot/feasibility study, the primary outcomes are feasibility and acceptability.

Primary outcomes include recruitment and retention rates, intervention completion rate and doula visit attendance; participant-reported experiences and acceptability (qualitative interviews and, if adopted, a satisfaction survey); and ART adherence and maintenance of viral suppression. Clinical and psychosocial measures are framed as exploratory signal-detection outcomes to inform a future multi-site trial (Aim 3).

Secondary outcomes include infant feeding method chosen and duration; breastfeeding complications (mastitis, cracked nipples, infant thrush, low milk supply); infant HIV testing completion and results; postpartum visit attendance; and changes in psychosocial measures (HIV stigma, medical mistrust, resilience).

### 2.10 Data analysis

**Quantitative analysis:** Descriptive statistics will characterize the study sample and summarize clinical outcomes. Metrics will include recruitment rate, retention rate, doula visit attendance, and intervention completion, with confidence intervals reported around key feasibility proportions (e.g., retention). As a pilot, the sample size (*N* = 15) is based on feasibility and resource considerations rather than a formal power calculation. Within-participant change over the study period (e.g., maintenance of viral suppression across pregnancy and the postpartum period, breastfeeding duration for those who elect to breastfeed, and infant HIV testing completion) will be summarized descriptively. Because this is a single-arm pilot without a control group, no between-group effect estimation or controlled pre-post inference is implied.

**Qualitative analysis:** Audio-recorded interviews will be transcribed and analyzed using thematic analysis with password-protected, cloud-based qualitative data processing systems (Dedoose). Analysis will employ line-by-line coding, constant comparison, and axial coding to identify central themes. CFIR constructs will be used as a deductive coding framework to evaluate implementation determinants (23, 24), supplemented by inductive codes emerging from the data. If both individual and group interviews are retained for participants (pending the decision in Section 2.6), they will be analyzed as separate data sources rather than pooled.

**Mixed-methods integration:** Quantitative and qualitative findings will be integrated using a convergent design, with results triangulated to provide a comprehensive understanding of intervention acceptability and preliminary effectiveness. Qualitative data from both Aim 1 (Advisory Group) and Aim 2 (participant interviews) will be used to refine the BRIDGES intervention for future testing.

## 3 Discussion

BRIDGES is the first HIV-specialized doula intervention supporting infant feeding choice in the United States. It fills the critical gap created by the 2023 guideline shift: shared decision-making for infant feeding among PPWH is now the standard of care, but no evidence-based support model exists to deliver it safely (6, 7, 10).

The intervention operationalizes several key guideline recommendations: patient-centered counseling beginning in pregnancy and continuing postpartum; multidisciplinary team coordination including lactation support; ART adherence support and viral load monitoring during breastfeeding; screening and support for postpartum depression; and nonjudgmental, culturally sensitive counseling that respects maternal autonomy (7, 10, 11, 30). By integrating an HIV-trained doula into the care team, BRIDGES extends the traditional doula scope of practice to include HIV-specific clinical knowledge (ART adherence, viral load monitoring, infant ARV prophylaxis) while preserving the doula's core role as a sustained, nonjudgmental source of emotional and informational support.

The intervention is innovative in three respects. First, it supports all infant feeding choices (breastfeeding, formula feeding, and banked donor milk) rather than privileging one option, consistent with the patient-centered approach recommended by current guidelines (7, 10, 11). Second, it embeds community engagement through the Advisory Group, ensuring that the intervention is informed by the lived experiences of people with HIV and the expertise of perinatal HIV providers. Third, the multilingual capacity (English, Spanish, Haitian Creole) and South Florida setting ensure representation of communities disproportionately affected by HIV, including Black, Hispanic/Latina, and Haitian Creole-speaking populations.

Doula care has demonstrated consistent benefits for breastfeeding initiation, maternal anxiety reduction, and postpartum follow-up, and has been identified as a promising approach to mitigate racial disparities in maternal-infant health outcomes (15–19). However, prior doula studies are limited by a lack of HIV-specific evaluation. The Kotawêw study in Canada represents the only published work examining doula care within the HIV context, describing Indigenous doulas as providing peer mentorship, practical HIV education, stigma reduction, and structural advocacy (21). BRIDGES builds on this foundation by developing a structured, replicable intervention model for the U.S. clinical setting.

Several limitations should be acknowledged. The single-arm design lacks a control group; however, this is appropriate for an initial evaluation of a novel intervention where the primary goal is to assess acceptability and generate preliminary data for a future controlled trial. The sample size (*N* = 15) limits statistical power; however, the mixed-methods design compensates by generating rich qualitative data on implementation determinants and participant experience. The single-site setting may limit generalizability; however, the UM Women's HIV Clinic serves a racially, ethnically, and linguistically diverse population representative of the communities most affected by HIV nationally. The 12-week postnatal follow-up may not capture the full duration of breastfeeding or longer-term outcomes; however, this window encompasses the critical period for breastfeeding establishment, early ART adherence challenges, and initial infant HIV testing milestones.

The implications of this work extend across multiple stakeholders. For clinicians, BRIDGES provides a replicable model for operationalizing the 2023 guideline shift through structured doula-mediated support. For PPWH, it offers sustained, culturally concordant support for autonomous infant feeding decisions in a clinical landscape where such support is largely absent. For policymakers, the expanding integration of doula care into Medicaid-covered perinatal services nationally (15, 20) creates a pathway for sustainable implementation of HIV-specialized doula programs. Future directions include a multi-site randomized controlled trial with longer follow-up, evaluation of cost-effectiveness, and adaptation of the BRIDGES model for telehealth delivery to reach PPWH in rural and underserved areas.

## 4 Ethics and dissemination

### 4.1 Ethics statement

The studies involving humans will be reviewed and approved by the University of Miami Institutional Review Board prior to enrollment. The studies will be conducted in accordance with the local legislation and institutional requirements. The study involves a vulnerable population (pregnant women) and will adhere to all applicable federal regulations for the protection of human subjects.

### 4.2 Informed consent

Patient participants (Aim 2) will provide written informed consent in a private room at the UM CFAR Clinical Research Unit. The research assistant will explain the study's purpose and protocol, read the informed consent document, and clarify any questions. Consent will be available in English, Spanish, and Haitian Creole. If a participant wishes to discuss participation with family members, a follow-up appointment will be scheduled. Advisory Board members (Aim 1) will provide verbal consent at the first Advisory meeting.

### 4.3 Confidentiality and data protection

Data and identifiers will be kept confidential and stored securely. Paper records will be locked in the P.I.'s office with access limited to approved study personnel. Electronic files will be password-protected in a UM-approved cloud storage system (Box). A coded identification system will be used, with the link to participant identity maintained separately from research data. Paper records will be destroyed within 3 years; electronic files will be deleted within 3 years. HIPAA authorization will be obtained from research subjects prior to data collection from the electronic medical record.

### 4.4 Privacy protections

The consent form clearly states participants' right to decline any question and to withdraw at any time without affecting their medical care at the Women's HIV Clinic. Given the sensitive nature of HIV status and infant feeding decisions, all study interactions will occur in private settings. No questions about immigration status will be asked. For any reporting to authorities, no mention of participation in this protocol will be disclosed.

### 4.5 Risks and benefits

Risks include breach of confidentiality and discomfort answering sensitive questions. These risks are minimized through the confidentiality protections described above and participants' right to skip questions or withdraw. There is no guaranteed direct benefit for participation. Potential indirect benefits include additional support from the doula, referrals to services, and enhanced monitoring of HIV treatment outcomes.

### 4.6 Participant compensation

Patient participants will receive a maximum of $210: $150 for data collection ($50/visit×3 visits) and $60 for transportation as shared ride service to research visits ($20/round-trip×3 visits). Advisory Group members will not receive compensation.

### 4.7 Dissemination

Results will be disseminated through peer-reviewed publications, scientific conferences, and community forums. The Advisory Group will participate in developing community-facing dissemination materials. The BRIDGES intervention manual and training materials will be made available for adaptation by other U.S.-based HIV clinics providing pregnancy care services to PPWH.

## Data Availability

The datasets generated and/or analyzed during this study will be available from the corresponding author upon reasonable request, subject to institutional review board approval and applicable data sharing policies.
