# Correction: BRIDGES: a patient-centered, community-informed HIV doula program that supports infant feeding choice among Hispanic and Caribbean families: a study protocol

**DOI:** 10.3389/frph.2026.1989121

**Published:** 2026-10-05

**Authors:** Rachel Mpanu-Mpanu, Melanie Francis, Kassandra N. Garcia, Lunthita M. Duthely

**Affiliations:** 1 Tamer Family Department of Obstetrics, Gynecology and Reproductive Sciences, University of Miami School of Medicine, Miami, FL, United States; 2 Ross University School of Medicine, Bridgetown, Barbados; 3 Department of Public Health Sciences, University of Miami School of Medicine, Miami, FL, United States

**Keywords:** breastfeeding, doula, HIV, infant feeding, maternal and child health

**Incorrect Funding**

In the published article, there was an error in the Funding statement. The funder, the University of Miami's Center for HIV and Research in Mental Health (CHARM), NIH Award P30MH133399, was erroneously omitted. The correct Funding statement appears below.

**Funding**

The author(s) declared that financial support was received for this work and/or its publication. This project was supported by the University of Miami's Center for HIV and Research in Mental Health (CHARM) through NIH Award P30MH133399. The content is solely the responsibility of the authors and does not necessarily represent the official views of the National Institutes of Health.

The authors apologize for this error and state that this does not change the scientific conclusions of the article in any way. The original version of this article has been updated.

